# 4437 The Prevalence of Food Insecurity Among University of Utah Medical Students: Documenting the Need for Supportive Programs

**DOI:** 10.1017/cts.2020.282

**Published:** 2020-07-29

**Authors:** Alyssa Thorman, Harneet Kaur Dhillon

**Affiliations:** 1 University of Utah School of Medicine; 2 University of Utah Medical School

## Abstract

OBJECTIVES/GOALS: Undergraduates experience food insecurity at rates 21% higher than the general population. Because professional students have been omitted from these studies, the goal of this project is to determine the prevalence of food insecurity among medical students at one academic institution. METHODS/STUDY POPULATION: A cross-sectional research design was used to quantify the food insecurity status of medical students at the University of Utah. The USDA’s validated 6-item Food Security Survey Module was distributed via email to all currently matriculated medical students. Student’s responses were anonymous but questions about gender and age were included. Respondents (N = 200) were scored per the module as food secure, food insecure, or very low food security. RESULTS/ANTICIPATED RESULTS: Statistical analysis included frequencies and chi-square tests. Medical students (N = 166) showed 50.6% of respondents experienced food insecurity in the past 12 months, 16.3% experienced very low food security.While there were no significant relationships between food security status and gender or age, general trends did show divorced and separated students had higher food insecurity risk 82%. A similar study in 2014 surveyed undergraduates at the same location; 51% of respondents (N = 221) experienced food insecurity. While medical students experience food insecurity at rates much higher than the national average, prevalence is lower than undergraduates at the same institution. DISCUSSION/SIGNIFICANCE OF IMPACT: Burnout and suicide in medical training are at an all-time high; professional and academic pursuits are limited when physiological needs of food security are not being met. Study results suggest, 50% of respondents are food insecure. This should inform the development of supportive programs.

